# A High-Finesse Suspended Interferometric Sensor for Macroscopic Quantum Mechanics with Femtometre Sensitivity

**DOI:** 10.3390/s24072375

**Published:** 2024-04-08

**Authors:** Jiri Smetana, Tianliang Yan, Vincent Boyer, Denis Martynov

**Affiliations:** 1 Institute for Gravitational Wave Astronomy, School of Physics and Astronomy, University of Birmingham, Birmingham B15 2TT, UK; tianliang@star.sr.bham.ac.uk (T.Y.);; 2 School of Physics and Astronomy, University of Birmingham, Birmingham B15 2TT, UK; v.boyer@bham.ac.uk

**Keywords:** interferometry, quantum optics, macroscopic quantum mechanics

## Abstract

We present an interferometric sensor for investigating macroscopic quantum mechanics on a table-top scale. The sensor consists of a pair of suspended optical cavities with finesse over 350,000 comprising 10 g fused silica mirrors. The interferometer is suspended by a four-stage, light, in-vacuum suspension with three common stages, which allows for us to suppress common-mode motion at low frequency. The seismic noise is further suppressed by an active isolation scheme, which reduces the input motion to the suspension point by up to an order of magnitude starting from 0.7 Hz. In the current room-temperature operation, we achieve a peak sensitivity of 0.5 fm/$\sqrt{\mathrm{Hz}}$ in the acoustic frequency band, limited by a combination of readout noise and suspension thermal noise. Additional improvements of the readout electronics and suspension parameters will enable us to reach the quantum radiation pressure noise. Such a sensor can eventually be utilized for demonstrating macroscopic entanglement and for testing semi-classical and quantum gravity models.

## 1. Introduction

Interferometric devices make excellent candidates for the probing of weak signals on the quantum scale. This is due to the impressive sensitivity achievable in such devices and their ability to form complex quantum systems.

Interferometers see widespread use in the development of laser technologies, atom trapping [1], and extend beyond mere photons with atom interferometry used in novel tests of fundamental physics [2,3]. Laser interferometers make particularly impressive sensors of displacement due to the sharp intensity response induced by microscopic (sub-wavelength) displacements of the key optical components. When one of the mirrors in the interference path is attached to a moving body, its relative displacement can be measured with excellent precision.

One of the most prominent uses of laser interferometry is in the kilometre-scale facilities for detecting gravitational waves (GWs). The Advanced LIGO (aLIGO) [4] and Advanced Virgo (AdV) [5] gravitational wave observatories currently serve as the gold standard for precision displacement sensing with aLIGO achieving a peak sensitivity of 2 × 10^−20^ m/$\sqrt{\mathrm{Hz}}$ during the most recent completed observing run (O3) [6]. Since the original detection of signal GW150914 [7], the detectors have observed dozens of sources [8,9,10]. Such measurements can offer us insight into aspects of general relativity [11], stellar and black-hole population statistics [12], and the structure of neutron stars [13], among other aspects. The role of interferometers in GW detection will only continue to expand following construction of next-generation terrestrial observatories (CE [14] and ET [15]), and the million-kilometre-scale LISA detector [16]. Interferometric detectors are seeing growing application in the search for dark matter particles [17,18,19], quantisation of spacetime [20,21], and entanglement [22], playing a pivotal role in the development of particle physics and cosmology into the future.

For the displacement-sensing laser interferometer, the quantum nature of light imposes an undesirable limitation to the detector’s sensitivity. Through the formalism of Caves and Schumaker [23,24], we can analyze the nature of quantum noise in reference to the two so-called quadratures of the electromagnetic field (amplitude and phase), which form a conjugate pair. The intrinsic and inescapable fluctuations in the two quadratures of light couple to the displacement measurement of the interferometer. In a simple Fabry–Perot interferometer, the phase quadrature fluctuations couple directly to the readout, resulting in the so-called quantum shot noise (QSN), whilst fluctuations in the amplitude quadrature induce a force on the cavity mirrors—the so-called quantum radiation pressure noise (QRPN) [25].

We write the total quantum noise in a suspended, simple Fabry–Perot cavity (zero end mirror transmission) following the conventions in Ref. [26] as

(1) $$S_{x}^{qn}\left(\Omega \right)=\left(\frac{1}{\kappa }+\kappa \right)\frac{S_{\mathrm{SQL}}\left(\Omega \right)}{2},$$

where κ is a frequency-dependent collection of terms given by

(2) $$\kappa =\frac{\omega_{0}T_{1}P_{c}}{mL^{2}\Omega^{2}\left(\Omega^{2}+{\left(\frac{T_{1}c}{4L}\right)}^{2}\right)},$$

and the common parameters are collected in $S_{\mathrm{SQL}}$, which is given by

(3) $$S_{\mathrm{SQL}}\left(\Omega \right)=\frac{8\hbar }{m\Omega^{2}}.$$

In the equations above, $\omega_{0}$ is the laser’s central angular frequency, $T_{1}$ is the power transmissivity of the input coupler, $P_{c}$ is the power circulating inside the cavity, *m* is the mass of the cavity mirror, *L* is the cavity length, and all other symbols retain their usual meaning. The derivation uses free mass approximation. Therefore, the equations are valid for suspended interferometers for frequencies $\Omega \gg \omega_{n}$, where $\omega_{n}$ is the highest resonance of the suspension. It is readily apparent from Equation (1) that the quantum noise is minimized when κ=1, which gives rise to the concept of the standard quantum limit (SQL) in Equation (3) as a bounding surface corresponding to the minimum noise level achievable in a given interferometer configuration. The SQL, as a trade-off between the light acting as an imperfect probe (QSN) and the light disturbing the probed system leading to back action (QRPN), was first formulated in Ref. [27]. An important observation is the simplicity of Equation (3), with the SQL boundary being only dependent on the mass of the cavity mirrors.

However, the SQL does not represent a fundamental quantum limit to any and all interferometric systems. The development of nonlinear optics has enabled us to manipulate the quantum noise components in the two quadratures in the form of optical squeezing—a technique that reduces the quantum noise in one quadrature at the expense of a rise in the other, first proposed in Ref. [28]. As the interferometer’s displacement signal is read out from only one quadrature, shifting the quantum noise from this quadrature to the other has the effect of reducing the measured quantum noise to a level below the nominal SQL defined above. Squeezing has been demonstrated across a range of experiments [29,30,31] and successfully applied in the current generation of GW detectors [32,33]. Other techniques have been proposed for manipulating and reducing quantum noise, such as phase-insensitive amplification via opto-mechanical coupling [34,35,36].

Whilst the theory of the quantum behaviour of light is well advanced, many aspects of the theory lack experimental verification. Crucially, no experiment has yet operated at the sensitivity level of the SQL, with the GW detectors unable to reach the SQL due to the coating thermal noise [6,37]. However, an interferometer operating at the quantum noise level over a broad band covering the SQL frequency would enable us to investigate novel aspects of quantum behavior, such as entanglement on the macroscopic scale [38], and allow for us to test the quantum nature of gravity [39,40].

In this paper, we present our table-top interferometeric sensor for probing macroscopic quantum mechanics and demonstrate a femtometre peak displacement sensitivity in the audio band. The experiment closely follows practices and experience adopted from the GW community motivated by the advanced noise suppression techniques implemented in GW detection and the near-SQL sensitivity of the detectors. The interferometer consists of a high-finesse suspended cavity in a cryogenic environment. Suspending the mirrors (a feature we share with GW detectors) is a departure from typical tabletop configurations but is beneficial for our purposes as the low resonant frequencies of the suspension chain increase the coupling strength of the QRPN to displacement, and thus allow for us to eventually reach the SQL.

We design the system to place the SQL frequency at 100 Hz, consistent with the aLIGO detectors, but raise the SQL level by reducing the mass of the cavity mirrors to 10 g. Further reduction in mass is not necessarily desirable due to the likely increase in the suspension resonances (cantilever microresonators, for example, can have a resonant frequency in the kHz to 100 s kHz [41]) and the increased control difficulty arising from Sigg–Sidles instability [42]. This mirror mass also has the benefit of corresponding to the typical mass of common 1-inch tabletop optics. A similar approach of using gram-scale mirrors in a suspended cavity to reach the QRPN is investigated in Ref. [43] and a large-scale experiment for reaching the SQL is currently undergoing commissioning at the Albert Einstein Institute (AEI) 10-metre prototype [44]. This work follows on from preliminary presentations in Refs. [45,46] and the design study in Ref. [47].

We explore in detail the key aspects of the design in Section 2, focusing on the optical system, the internal suspension and the active isolation system. In Section 2.4, we present the sensitivity of the sensor together with a discussion of the noise budget and future steps towards reaching QRPN-dominated operation—the next major milestone towards realizing a full quantum sensor.

## 2. Experimental Layout

The two key noise sources that limit such systems from reaching the SQL are the thermal noise and the seismic noise. The latter is mitigated by the choice to suspend the cavity with a sufficiently soft multi-stage suspension. To this end, we have the examples from across the GW community to draw upon (see Section 2.2). The thermal noise is the most challenging noise source to mitigate, best reflected by the aLIGO and AdV detectors, which, whilst close to the SQL, are currently dominated at the SQL frequency by the coating thermal noise [6,37].

Via the fluctuation–dissipation theorem [48,49,50], we can write the thermal noise of a generic mechanical system [51] as

(4) Sxth(Ω)=4kBTRe(Y(Ω))Ω2,

where Y(Ω) is the mechanical admittance that contains the dissipative element of the system’s response. The most direct strategy for reducing thermal noise, as expressed in Equation (4), is simply to reduce the temperature of the system; hence, we design our experiment around the constraints of a cryostat to enable future cryogenic operation. Aside from temperature, the specifics of the system’s response are crucially important and the key component of thermal noise mitigation is the appropriate choice of material based on desirable mechanical and thermal properties. Our approach shares some of the cryogenic challenges with the GW detector, KAGRA [52], and prospective next-generation detectors, LIGO Voyager [53], and ET, which we draw upon in our design.

**Figure 1:**
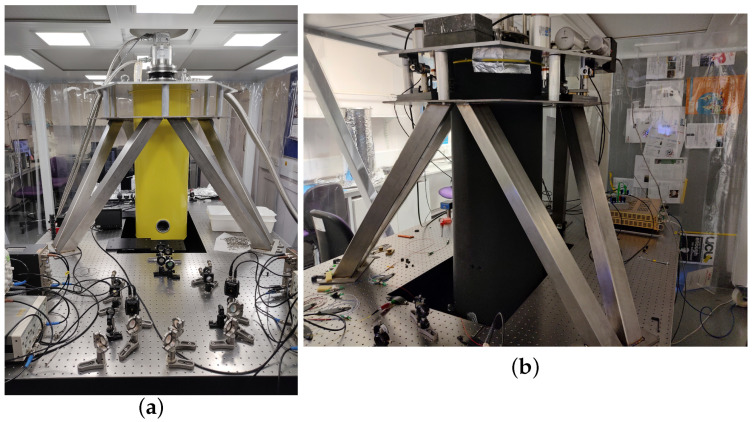
Panel (**a**) shows the experimental set-up in an earlier iteration without active seismic isolation of the cryostat. This image shows the key optical and electronic components, as well as the exposed vacuum lid showing the location of the viewports. Panel (**b**) shows the same experiment in its current status. The parallel plates near the top of the cryostat support an array of seismometers, coil–magnet actuators, passive rubber springs and counterweights. In this image, many electronic components are moved and the cryostat lid is wrapped in soft foam, thus obscuring most of the viewport’s aperture. These latter changes are implemented to address acoustic noise that we discovered coupled into some areas of the detection band.

One important consequence of cryogenic operation is the increased difficulty of implementing active components. Even at the current room-temperature stage, we design the optical and mechanical system without any in-vacuum suspension sensing or active control. If we can achieve low-noise operation in this regime, we will avoid significant technological challenges down the line. See Figure 1.

### 2.1. Optical System

**Figure 2:**
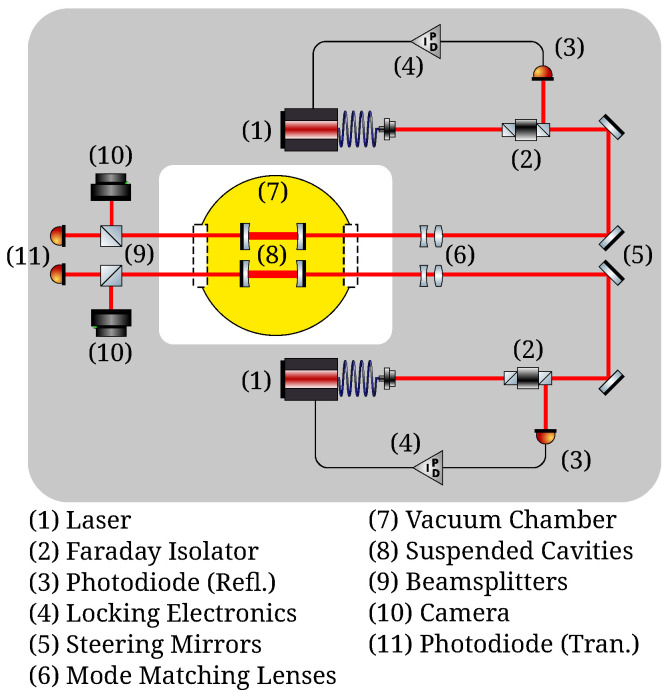
Optical layout of the experiment showing the two identical interferometric systems and the suspended cavities inside the vacuum envelope. Figure reproduced from Ref. [47].

The optical layout is duplicated into two identical systems, each based around an independent, suspended Fabry–Perot optical cavity. The cavity mirrors are the only optical components that require cooling and isolating and are, therefore, the only optical components inside the vacuum envelope. The optical layout is shown in Figure 2.

We currently use fused silica mirror substrates in the room-temperature iteration of the experiment due to their good optical and mechanical properties (low absorption [54], loss angle 10^−7^ [55]). Silica performance degrades at low temperatures due to the loss peak of 10^−3^ [56] around 20 K, making its use unsuitable for later cryogenic operation. In the future, we plan to transition to silicon substrates due to the superior mechanical properties—loss angle of 10^−7^ [57]. Its worse thermoelastic noise due to the relatively high coefficient of thermal expansion is mitigated by appropriately tuning the operating temperature near the critical temperature of 20 K where the thermal expansivity crosses zero [58].

The dielectric coatings we use currently are composed of Ta_2_O_5_/SiO_2_ layers deposited by ion beam sputtering [59]. These are chosen due to their high quality (low optical losses [60] and loss angle in the range 1 × 10^−4^ to 4 × 10^−4^ [61]) and proven track record in precision interferometry. In preparation for the switch to silicon substrates, we also consider other coating options more suitable for cryogenic operation. We currently consider amorphous—a-Si (amorphous silicon)/SiO_2_, a-Si/SiN–or crystalline—GaAs/AlGaAs—candidates.

We measure the bandwidth (FWHM) to be 4020 Hz for a cavity length of 95 mm. This yields a measured finesse of 3.9 × 10^5^. The measured transmissivity of the mirrors is $7.5_{-1.5}^{+1.0}$ ppm. From the measured finesse, we determine the total round-trip losses to be approximately 16 ppm, which suggests that the additional cavity losses on top of the transmissivity do not exceed more than a few ppm. However, due to the difficulty of measuring the individual loss components (including the mirror transmissivity) to sub-ppm precision, we cannot provide a comprehensive breakdown of each source of loss. Our estimate of the cavity loss is generally in agreement with the study in Ref. [62], which reviews the realistic prospects for low-loss optical cavities of different lengths. This results in a high build-up of intra-cavity power, which amplifies the QRPN coupling without the need for high input power. Such high finesse coupled with the softly suspended and light cavity mirrors presents significant challenges to controllability.

The combined challenge of the above factors is most noticeable during the initial lock acquisition. We make use of a Pound–Drever–Hall locking scheme [63], with sidebands at 4 MHz. Due to the lack of active cavity control, we are limited to locking via laser-frequency actuation. We use a Rio Orion 1550 nm, 24 mW distributed feedback laser with two means of frequency control: the slow but large range control of the laser temperature, and the fast but low-range modulation of the laser current supply. The current controller is further divided into a slow DC controller with DC range of 800 MHz frequency tuning over 8 Vpp, and an AC-coupled fast controller with a peak response at 20 kHz, continuing into 10s MHz, and a range of 400 MHz frequency tuning over 1 Vpp. Due to the short cavity length, the cavity free spectral range (FSR) is 1.67 GHz, which is outside of the current controller range and makes lock acquisition more challenging.

The resonant condition is maintained via feedback to the two supply current channels with a servo bandwidth of 300 kHz. This is necessary, as the high finesse leads to a narrow error signal, which, combined with the relatively high laser frequency noise of 10 Hz/$\sqrt{\mathrm{Hz}}$ at 1 MHz, places strict requirements on the servo bandwidth. The temperature setpoint can be tuned periodically by hand to compensate for accumulated laser frequency drifts on the week time scale.

Another challenge of the lock acquisition process is the initial buildup of intra-cavity power. This generates strong classical radiation pressure, which causes a large drift in the cavity length due to the soft suspension and light cavity mirrors. With power in excess of 0.5 mW, this shift is beyond the range of the current controller and the cavity drifts out of lock. Our current strategy for achieving lock is through the use of a variable optical attenuator (VOA). The VOA allows us to maintain low input power initially, which reduces the magnitude of the rapid radiation pressure buildup during initial lock. Once a stable lock is established, we can increase the input power slowly whilst compensating for the frequency shift with the laser temperature to keep within the range of the current controller.

We set up two identical systems and beat the two independent lasers together to form an additional readout channel. This is necessary as we require a readout channel that is free of the laser frequency noise, which lies six orders of magnitude above the nominal SQL sensitivity at 100 Hz. The issue could be resolved with a single cavity and a low-noise frequency reference, but this is a challenge given our strict noise requirements at 100 Hz. The aggressive seismic noise suppression above 10 Hz coupled with the laser-frequency locking to the cavity motion means that the laser phase is very stable in the key frequency band around 100 Hz. The cavity can, in effect, act as a high-quality frequency reference in a limited band. Introducing a second cavity with very similar noise characteristics allows for us to use one cavity as the frequency reference for the other.

We can tune the central frequency of the beat-note by shifting the lock-point of each cavity by integer multiples of the FSR to a coarse value around 150 MHz. We then measure the frequency drift of this beat-note with a custom-built phase lock loop (PLL) based around a Minicircuits JCOS-175LN (Minicircuits, New York City, NY, USA) voltage-controlled oscillator (VCO). The key challenge here is one of dynamic range. Whilst the two cavities are quiet within the frequency band of interest, they suffer from high root mean square (RMS) motion, which comes almost entirely from the seismically excited mechanical resonances of the suspension chain. In order to achieve a sufficiently low electronics noise of the PLL, we use a lower VCO frequency range, which reduces the maximum tolerable cavity RMS motion. The natural RMS motion is larger than this VCO range, which means that we cannot reach our noise requirements with a purely passive seismic isolation system. Our solution involves the active inertial control of the cryostat, which is discussed in more detail in Section 2.3.

The locking procedure is entirely achieved using analogue electronics. However, we digitize the control signals for processing using the Control and Data System (CDS) used in aLIGO. The input signals are digitized at 64 Hz by a 16-bit, 20 Vpp analogue-to-digital converter (ADC) via an input anti-aliasing filter with a cut-off frequency at 7 kHz. This allows for us to easily search for coherence between different signals during commissioning and is also used to shape the control signals of the low-bandwidth active isolation feedback loops.

### 2.2. Suspension

Seismic noise comprises one of the principal sources of noise in suspended interferometers and is generally the dominant noise source at low frequencies. The conventional means of suppressing this noise within the GW community is to softly suspend the optics to achieve a sufficient level of passive isolation. Some different approaches to high-performance suspension design can be seen in the aLIGO quadruple suspension [64], the AdV superattenuator [65], and the predecessor GEO600 suspension [66].

To achieve horizontal isolation, we use a four-stage pendulum chain formed of intermediate aluminium masses and steel wire connections. The suspension is attached to the 10 K cold plate inside the cryostat to facilitate conductive cooling of the suspension and cavity optics in the future. Aluminium is chosen for its light weight (see below) and good thermal properties, particularly the impressive thermal conductivity of high-purity aluminium (in excess of 10^4^ W m^−1^ K^−1^ at cryogenic temperatures [57]). The steel wires are an intermediate step for room-temperature operation. The poor conductivity and relatively high loss angle of steel makes it unsuitable for cryogenic operation for both cooling and thermal noise considerations. Fused silica and silicon fibers are possible candidates for later cryogenic operation due to their low mechanical loss, whilst beryllium copper or tungsten show promise from a thermal conductivity perspective. The upper three stages are suspended over a length of 19 cm each with the cavity mirrors suspended by a shorter two-wire pendulum of length 2 cm.

For vertical isolation, we use triangular blade springs. The blades have a base width of 3 cm and a length of 130 cm and 200 cm for the top stage and intermediate stages, respectively. The thicknesses are tuned to lower all vertical resonances below 10 Hz. The intermediate stage blades are arranged along the diameters of their respective stage masses to maximize the length, as a longer and thicker blade achieves a lower resonant frequency than a shorter and thinner blade given equal internal stress (Section 6.4 in [67]). There are three vertical stages (one fewer than horizontal) on account of the stronger direct coupling of horizontal motion to the cavity length in contrast to the lower cross-coupling of vertical-to-horizontal motion. We currently use 316 stainless steel due to availability limitations, which has a comparatively low yield strength of around 200 MPa compared to the potential yield strengths in excess of 1 GPa of numerous maraging steels [68]. With a better choice of material in the future, it will be possible to lower all vertical resonances to below 3 Hz.

We use eddy current damping [69] between the upper intermediate and penultimate masses to avoid bypassing the upper two stages of isolation. The purpose of the damping is to reduce the Q factors of the suspension resonances and thus reduce the RMS motion and ease the range requirements on the PLL outlined in Section 2.1. For efficient damping of all resonances from a single stage, it is important to ensure all stages are well coupled to each other. This is achieved by making all stages comparable in mass and length, which is difficult to realize given the fixed 10 g mass of the mirrors. As such, the passive damping is only a partial solution to the RMS problem. Alternative damping solutions exist, such as active damping as with the Birmingham optical sensor and electromagnetic motor (BOSEM) [70] in aLIGO which offer certain advantage such as preventing a factor of *f* loss of suppression at high frequencies. We, however, continue to avoid active in-vacuum components unless deemed absolutely necessary.

**Figure 3:**
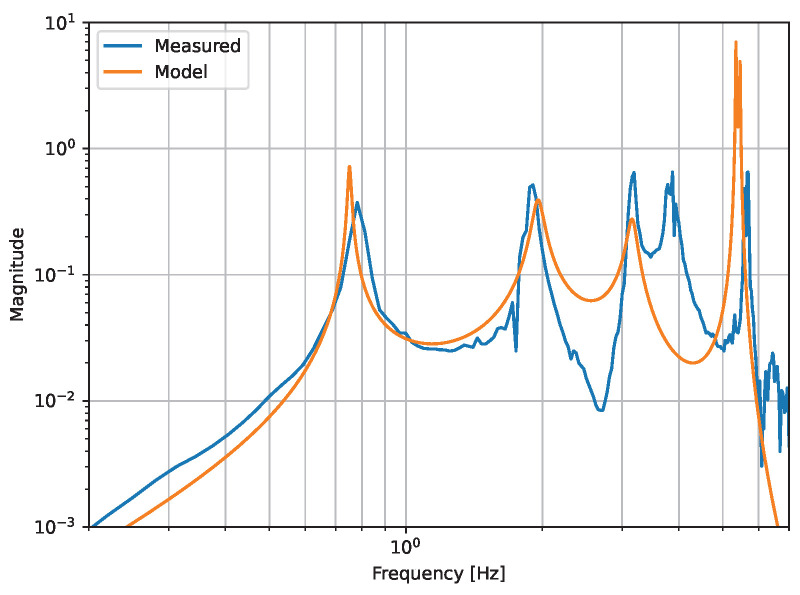
Transfer function of the suspension-point motion to the differential cavity motion. The Lagrangian model uses independently obtained values for the suspension parameters and only contains one free parameter (aside from the Q of the resonances), which is the unknown mismatch between the wire stiffness of the two final mirror stages. The model can be further improved by constraining the clamping losses and other damping mechanisms to better predict the resonance Q factors, and by including the coupling between the two separate cavities (the current model only considers a single cavity) which would likely allow for us to explain the zero in the transfer function at 2.7 Hz. The modeled transfer function is shifted vertically to line up with the measured data for visual clarity. The true gain of the transfer function is determined using parameters that are difficult to measure independently and is thus unknown. However, the location of the resonances and the overall shape of the transfer function is independent of the overall gain, and these match the measured data with reasonable accuracy. The unknown resonance at approximately 3.8 Hz is thought to be a pitch resonance that is cross-coupled to the the longitudinal mirror displacement.

An important aspect of the suspension design is that both cavity mirrors are suspended from the same common penultimate mass. This provides common-mode rejection of seismic noise in the cavity displacement spectrum below the final mirror-stage resonances, as the cavity is only sensitive to differential motion. This leads to an $f^{2}$ suppression of seismic noise towards DC below the first suspension resonance. The gain of the suspension-point-to-differential-cavity transfer function is proportional to the mismatch between the stiffness of the two final mirror stages, meaning it is important to ensure the two suspensions are as similar as possible. In order to minimize the mass of the intermediate stages (for efficient damping) but maximize the moment of inertia to reduce angular resonances, we arrive at a ring-like structure. The full suspension schematic is shown in Figure 3.9 of Ref. [47]. The suspension model is based on a Lagrangian analysis, which is also presented in detail in Ref. [47]. In Figure 3, we show the transfer functions of displacement at the suspension point to differential cavity displacement for the principal cavity axis-aligned horizontal degree of freedom (DoF, referred to in this case as the X DoF). This figure contains the measured transfer function, achieved by exciting the X DoF with the active isolation’s actuators (see below) and witnessing with the cavity-locking control signal. The measured transfer function is compared with the result of the Lagrangian analysis and correctly reproduces the overall shape of the transfer function, including the gain and the location of the resonances.

### 2.3. Active Isolation

The beat-note RMS must be reduced to below the VCO range of 50 MHz, which we cannot achieve with passive internal isolation alone. As the RMS is dominated by seismic coupling to the cavity lengths, we equivalently need to reduce the RMS seismic noise to significantly below 20 nm. We achieve this by actively controlling the cryostat motion. This technique of inertial isolation is widely used within the GW community. We adopt principles shared with the control of Stewart platforms [71], inertial sensors, such as the 6D six-axis seismometer [72], and the high-performance isolation provided by the internal seismic isolation (ISI) of aLIGO [73].

The general scheme consists of an inertial sensor, actuator, and a sufficiently mechanically compliant support structure. Sensing is achieved with six single-axis L-4C geophones (three horizontal and three vertical). The signals from the spatially distributed and strategically oriented geophones can be combined to yield a diagonalized measurement of the full six DoFs (three linear and three angular).

The mechanical structure consists of a rigid cryostat frame, the 140 kg cryostat payload (of which the internal suspension and cavity comprises a negligible amount), and a set of three natural-rubber cone feet that the payload rests upon. The truncated rubber cones are 50 mm and 37 mm in diameter at the base and the top, respectively, with a height of 62.5 mm. This follows a similar strategy to the use of Viton support feet demonstrated in the passive isolation of a multi-stage optical table [74]. The relatively low Young’s and shear modulus of rubber leads to low resonant frequencies in all degrees of freedom, with horizontal resonances of 3.9 Hz and a vertical resonance at 7 Hz. Such a soft system would not be suitable for our use in a purely passive capacity. Whilst the low resonant frequencies improve suppression of seismic vibrations from the ground, they also increase the mechanical admittance to the force generated by the pulsing cryopump, which degrades the overall performance during cryogenic operation. The benefit of the active system is that the cryostat is inertially isolated from vibrations coupling through both channels.

We actuate on the cryostat relative to the rigid cryostat support frame with coil–magnet actuators derived from the BOSEM (sensing components removed). The wire coils have a resistance of 41.4 Ω and an inductance of 17.8 mH.

**Figure 4:**
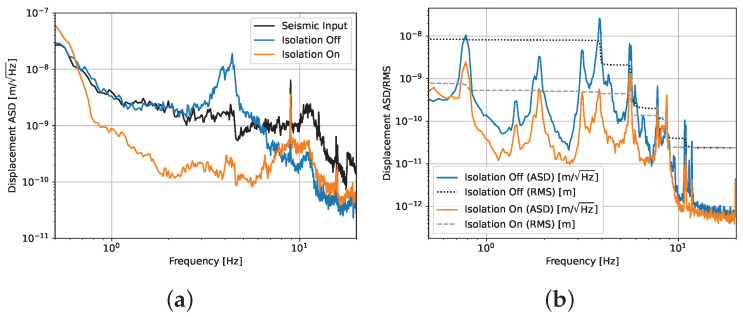
Panel (**a**) shows the performance of the active seismic isolation as seen by the geophones in the X DoF. The isolation off trace clearly shows the presence of the horizontal resonance of the rubber supports at 3.9 Hz and the passive suppression effect that is generated above this frequency. The isolation on trace shows an order-of-magnitude level of suppression compared to the input seismic motion and significant improvement over the passive-only effect in the region below 8 Hz. The isolation on trace inherits the passive isolation of the rubber springs above 8 Hz. Panel (**b**) shows the suppression of the seismic noise by the active isolation as witnessed by the cavity control signal. The RMS curves show the cumulative RMS integrated from high to low frequencies. The comparison of the isolation on/off curves shows a consistent reduction in the displacement spectrum with that measured by the in-loop geophones in Figure 4a. Importantly, the cavity sees an order-of-magnitude drop in its RMS motion when the active isolation in engaged.

We make use of the aforementioned CDS for data acquisition and control, which enables us to design a custom digital servo for the feedback control and make coherence measurements with the cavity length readout and obtain an out-of-loop witness channel. This allows for us to verify that the inertial isolation of the cryostat is truly leading to a reduction in the seismic coupling to the cavity motion. We construct a feedback servo, which leads to an order-of-magnitude reduction in the RMS displacement of the X DoF (see Figure 4a). This is reflected in a corresponding order-of-magnitude reduction in the differential cavity motion (Figure 4b). This suppression leads to a reduction in the RMS cavity displacement to below 1 nm, which is well within the range of the VCO with a sufficient safety margin.

### 2.4. Results

**Figure 5:**
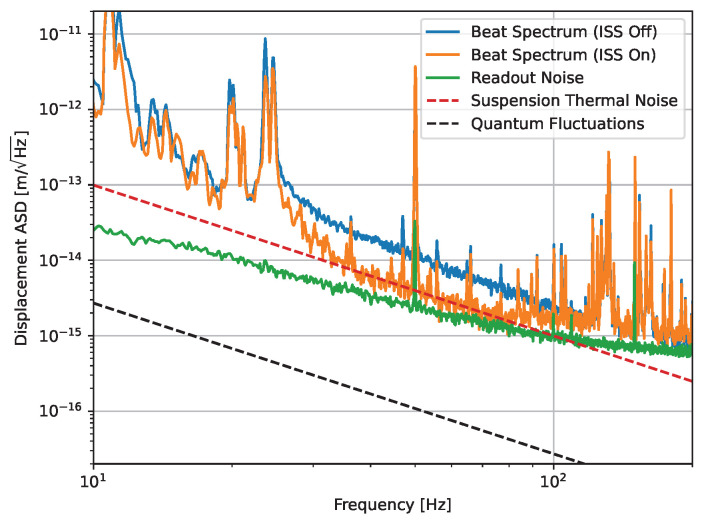
Plot showing the beat spectrum between the reflected fields from the two cavities viewed within the detection band. In the middle band from 30 Hz to 100 Hz, we observe the effects of laser intensity noise coupling via the classical radiation pressure effect shown in the ISS off trace. This noise is stabilized by the ISS down to a combination of the PLL readout noise in the upper region of the detection band, and the suspension thermal noise in the middle region. In the region below 30 Hz, we see the seismic noise growing rapidly towards the resonances below 10 Hz, which are shown in Figure 4b.

We show the amplitude spectral density of the beat between the reflected fields from the two cavities in Figure 5. The spectrum is shown in two configurations: one with intensity stabilization servo (ISS) off and one with ISS engaged. In the simplified treatment of the interferometric system, intensity fluctuations couple into the phase-sensitive beat readout as a second-order effect. However, the light cavity mirrors lead to a large mechanical admittance to the classical radiation pressure fluctuations caused by the intensity noise. We show that the suppression of this effect leads to an improvement by up to a factor of five in the frequency band from 30 Hz to 100 Hz.

The seismic and suspension thermal noise is sharply suppressed above the suspension resonances, leaving a broad region from 40 Hz onwards that is dominated by the readout noise. This noise is shaped by an analogue whitening filter to selectively reduce the readout noise in this critical band without reducing the range at low frequencies, which is needed to handle the high RMS motion arising from the seismic peaks. Currently, the readout noise is limited by the ADC noise. If we successfully improve on the ADC noise, we can achieve a factor of five reduction in the readout noise before we become limited by the PLL noise instead.

The key issue with reducing readout noise further is the large RMS in the ‘quiet’ band arising from broad peaks in the 200 Hz to 400 Hz region. The position of these peaks within the region amplified by the whitening filter means that we saturate the ADC if we attempt to amplify the input and reach the PLL noise. We identify these peaks as acoustic noise coupling to the vibrations of the view-ports of the cryostat’s vacuum lid. We mitigate this noise source by minimizing acoustic noise in the immediate experimental environment and by wrapping the cryostat in acoustically isolating foam. Whilst we are able to reduce this noise by approximately an order of magnitude compared to the original setup, additional isolation is necessary to reduce the RMS from this region and allow for us to reduce the ADC noise further.

At the current level of performance, we are capable of reaching a peak sensitivity of 0.5 fm/$\sqrt{\mathrm{Hz}}$ across a broad region in the acoustic frequency band. According to the projected noise budget for ultimate room-temperature operation (Figure 4.7 in Ref. [47]), we need to achieve a sensitivity of approximately 0.01 fm/$\sqrt{\mathrm{Hz}}$ at 100 Hz to reach QRPN-dominated operation and observe the quantum back-action effect in the suspended cavity. At this particular frequency, representing the key frequency of interest for our future SQL measurement that we show in Figure 5, we reach a sensitivity of 2 × 10^−15^ m/$\sqrt{\mathrm{Hz}}$.

As a point of comparison, the best sensitivities in suspended interferometers have been demonstrated in large-scale facilities. For example, during the O3 observing run, aLIGO demonstrated a sensitivity of 2 × 10^−20^ m/$\sqrt{\mathrm{Hz}}$ at 100 Hz in their 4 km interferometer [6]. Recent results from the AEI 10 m prototype show a sensitivity of 1 × 10^−17^ m/$\sqrt{\mathrm{Hz}}$ [75] at 100 Hz. In a more comparable experiment consisting of light optics in a table-top set-up, a sensitivity at 100 Hz of 2 × 10^−12^ m/$\sqrt{\mathrm{Hz}}$ was demonstrated in the suspended Michelson interferometer of Ref. [76] and 4 × 10^−16^ m/$\sqrt{\mathrm{Hz}}$ was demonstrated in the Fabry–Perot Michelson interferometer in Ref. [43]. In many of these cases, the sensitivity continues to improve towards higher frequencies (e.g., Ref. [43] reaches 2 × 10^−17^ m/$\sqrt{\mathrm{Hz}}$ at 1 kHz), which is due to their dominant noise source, thermal noise, improving towards higher frequencies, whilst our device is currently limited by the flat noise profile of the readout electronics above 100 Hz.

## 3. Conclusions

The quantum nature of light, coupled with the excellent sensitivity of modern interferometric devices, opens the door to the development of novel sensors for probing quantum mechanics on the macroscopic scale. In this paper, we present our development of a table-top quantum sensor with femtometre sensitivity within the acoustic frequency band.

Our sensor utilizes the expertise developed within the gravitational-wave community, particularly in the control of high-finesse, suspended interferometers, and the mitigation of seismic and thermal noise. We construct a system consisting of a cryostat-mounted four-stage suspension supporting two cavities with finesse in excess of 10^5^. We use active isolation of the entire cryostat system to achieve an order-of-magnitude of reduction in the seismic noise prior to the passive isolation of the internal suspension. We achieve the femtometre sensitivity using a custom-made phase lock loop.

The sensor in its current iteration serves as the first sensitivity milestone towards reaching the standard quantum limit. The ultimate low-noise mode is achieved during cryogenic operation, which requires significant changes to the materials to ones more appropriate for good low-temperature performance. The immediate next step is to reach the sensitivity of 1 × 10^−17^ m/$\sqrt{\mathrm{Hz}}$ at 100 Hz by achieving better readout noise, which, in part, involves the mitigation of ambient acoustic noise.

On a longer time scale, we prepare for cryogenic operation by switching to more appropriate materials. For the suspension, this involves making use of materials with much larger thermal conductivity, such as high-purity aluminium, tungsten, or copper. A major change to the optical system is the switch to silicon resonators with novel but less well-developed optical coatings. With these changes, we predict that it is possible to reach the standard quantum limit within the same acoustic frequency band presented in this paper.

## Data Availability

Data are contained within the article.

## Abbreviations

The following abbreviations are used in this manuscript:

ADC	Analogue-to-digital converter
AdV	Advanced Virgo
aLIGO	Advanced LIGO
BOSEM	Birmingham optical sensor and electromagnetic motor
CDS	Control and data system
DoF	Degree of freedom
FSR	Free spectral range
GW	Gravitational wave
ISI	Internal seismic isolation
ISS	Intensity stabilisation servo
PLL	Phase lock loop
QRPN	Quantum radiation pressure noise
QSN	Quantum shot noise
RMS	Root mean square
SQL	Standard quantum limit
VCO	Voltage-controlled oscillator
VOA	Variable optical attenuator
